# Interaction between epidermal growth factor receptor and C-C motif chemokine receptor 2 in the ovulatory cascade

**DOI:** 10.3389/fcell.2023.1161813

**Published:** 2023-04-04

**Authors:** J. G. Conte, M. L. Tellechea, B. Park, M. G. Ballerini, G. Jaita, M. C. Peluffo

**Affiliations:** ^1^ Centro de Investigaciones Endocrinológicas “Dr. César Bergadá” (CEDIE), CONICET—FEI—División de Endocrinología, Hospital de Niños Ricardo Gutiérrez, Buenos Aires, Argentina; ^2^ Instituto de Investigaciones Biomédicas (INBIOMED), Facultad de Medicina CONICET- Universidad de Buenos Aires, Buenos Aires, Argentina; ^3^ Biostatistics Shared Resource, Knight Cancer Institute, Oregon Health and Science University, Portland, OR, United States; ^4^ Departamento de Biología Celular e Histología, Facultad de Medicina-Universidad de Buenos Aires Buenos, Buenos Aires, Argentina

**Keywords:** epidermal growth factor receptor, C-C motif chemokine receptor 2, monocyte chemoattractant protein 1, cumulus-oocyte complex, feline, ovulatory cascade genes, cumulus-oocyte expansion, oocyte nuclear maturation

## Abstract

The epidermal growth factor receptor (EGFR) signaling pathway is one of the main pathways responsible for propagating the luteinizing hormone (LH) signal throughout the cumulus cells and the oocyte. Recently, we have proposed the C-C motif chemokine receptor 2 (CCR2) and its main ligand (monocyte chemoattractant protein-1, MCP1) as novel mediators of the ovulatory cascade. Our previous results demonstrate that the gonadotropins (GNT), amphiregulin (AREG), and prostaglandin E2 (PGE2) stimulation of periovulatory gene mRNA levels occurs, at least in part, through the CCR2/MCP1 pathway, proposing the CCR2 receptor as a novel mediator of the ovulatory cascade in a feline model. For that purpose, feline cumulus-oocyte complexes (COCs) were cultured in the presence or absence of an EGFR inhibitor, recombinant chemokine MCP1, and gonadotropins [as an inducer of cumulus-oocyte expansion (C-OE), and oocyte maturation] to further assess the mRNA expression of periovulatory key genes, C-OE, oocyte nuclear maturation, and steroid hormone production. We observed that MCP1 was able to revert the inhibition of *AREG* mRNA expression by an EGFR inhibitor within the feline COC. In accordance, the confocal analysis showed that the GNT-stimulated hyaluronic acid (HA) synthesis, blocked by the EGFR inhibitor, was recovered by the addition of recombinant MCP1 in the C-OE culture media. Also, MCP1 was able to revert the inhibition of progesterone (P4) production by EGFR inhibitor in the C-OE culture media. Regarding oocyte nuclear maturation, recombinant MCP1 could also revert the inhibition triggered by the EGFR inhibitor, leading to a recovery in the percentage of metaphase II (MII)-stage oocytes. In conclusion, our results confirm the chemokine receptor CCR2 as a novel intermediate in the ovulatory cascade and demonstrate that the EGFR/AREG and the CCR2/MCP1 signaling pathways play critical roles in regulating feline C-OE and oocyte nuclear maturation, with CCR2/MCP1 signaling pathway being downstream EGFR/AREG pathway within the ovulatory cascade.

## Introduction

The domestic cat (*Felis catus*) is a valuable model to study oocyte biology along with diverse infertility syndromes in women due to the highly conserved reproductive mechanisms between human and feline species (Wildt et al., 2010). Interestingly, cat oocytes share several characteristics with human oocytes (Combelles et al., 2002; Comizzoli et al., 2003) in contrast to typical laboratory mouse models, such as the diameter of the oocyte proper and the germinal vesicle (GV), the time to reach the MII-stage of meiosis in culture, and a nuclear configuration with a small nucleolus and fibrillar chromatin. In addition to the potential to expand knowledge of feline reproduction, the use of this model system has the advantage of providing an excellent surrogate for understanding events involving human COC that are necessary for fertility. In our laboratory, we have established two different feline culture systems, showing that the culture of feline antral follicles and/or their COCs was a robust and valuable system to study follicular development, steroidogenesis, periovulatory events, as well as follicle and COC biology in general (Rojo et al., 2015; Rojo et al., 2018; Jaworski et al., 2020).

Before ovulation, the LH surge triggers key processes for fertility, such as C-OE, resumption of oocyte meiosis, and rupture of the follicle wall. Oocyte nuclear maturation consists of the resumption and completion of the first meiotic division until arrest at the second meiotic metaphase, which is easily observed (Eppig and O’Brien, 1996). However, the oocyte only acquires full developmental competence when nuclear, as well as cytoplasmic maturation, are closely integrated. Cytoplasmic maturation encompasses the processes that enable the oocyte to be fertilized successfully and support embryo development but it is more difficult to assess (Zheng, 2007). Although these two programs can also proceed as independent processes to some extent (Sun and Moor, 1991). Meanwhile, the LH surge triggers another critical process known as C-OE. This process is a consequence of the loss in cell-to-cell contacts and an HA-rich extracellular matrix (ECM) being synthesized, mainly by HA synthase-2 (HAS2), resulting in a large increase in area or expansion of the cumulus granulosa cell layer that surrounds the oocyte (Chen et al., 1993; Fulop et al., 1997; Hess et al., 1999; Kimura et al., 2002; Salustri et al., 2004). This HA-rich-containing matrix is composed and stabilized by several plasmas- and follicle-derived components (Richards, 2005) including inter-alpha trypsin inhibitor (IαI) and associated heavy chains (HC1–3), proteoglycans such as versican, the tumor necrosis factor alpha-induced protein 6 (TNFAIP6), pentraxin 3 (PTX3), as well as certain members of the a-disintegrin and metalloproteinase with thrombospondin-like repeats (ADAMTS) family of proteases (Chen et al., 1992; Mukhopadhyay et al., 2001; Fulop et al., 2003; Russell et al., 2003; Salustri et al., 2004; Richards, 2005).

While some of the autocrine and paracrine factors important for the periovulatory events have been identified, the molecular mechanisms responsible for initiating such complex processes are not fully understood. During the periovulatory interval, the activation of a complex signaling network is essential to induce and guarantee LH-dependent processes since the expression of the LH receptor is mainly restricted to the mural granulosa cells (Peng et al., 1991). One of the main pathways responsible for propagating the LH signal throughout the cumulus cells and the oocyte is the system composed of the EGFR and its ligands (such as AREG, epiregulin (EREG), and betacellulin (BTC)) (Park et al., 2004; Richani and Gilchrist, 2018). Indeed, it was demonstrated that EGF-related factors mediate the gonadotrophin action through the induction of steroid and prostaglandin production (Jamnongjit et al., 2005; Shimada et al., 2006). On the other hand, we have recently proposed the chemokine receptor CCR2 and its main ligand (MCP1) as novel mediators of the ovulatory cascade, demonstrating that MCP1 increases the mRNA levels of key genes involved in the ovulatory cascade [*HAS2*, *AREG*, *TNFAIP6*, *PTX3*, and growth differentiation factor-9 (*GDF9*)] within the feline COC *in vitro* (Rojo et al., 2019). And interestingly, in our previous work when the CCR2 receptor was inhibited in the COC *in vitro*, the stimulation of mRNA levels of different periovulatory genes (*AREG*, *HAS2*, and *TNFAIP6*) triggered by recombinant AREG was repressed (Jaworski et al., 2020), suggesting that MCP1/CCR2 system may be downstream the EGF-related factors in the ovulatory cascade. Supporting this idea, studies on MCP1 stimulation by EGF-like ligands have been reported in other tissues (Zhu et al., 2007; Liu et al., 2008).

Both types of receptors—G protein-coupled receptors (GPCRs; such as CCR2) and receptor tyrosine kinases (RTKs; such as EGFR)—share common signaling intermediates in the pathway (Luttrell et al., 1999). Although an increasing number of studies show that cross-communication can be generalized to several GPCRs and diverse cell types, between GPCRs and EGFR (Eguchi et al., 1998), the biological significance of this crosstalk within the COC remains unknown, and thus further studies are warranted. Therefore, the aim of our study was to evaluate the participation and interactions in the ovulatory cascade of the EGFR/AREG and the CCR2/MCP1 signaling pathways, using the domestic cat as our model. For that purpose, feline COCs were cultured in the presence or absence of an EGFR inhibitor, recombinant chemokine MCP1, and GNTs (as an inducer of C-OE and oocyte maturation) to further assess the mRNA expression of periovulatory key genes (*AREG*, *HAS2*, *TNFAIP6*, and *PTX3*), C-OE, oocyte nuclear maturation, and steroid hormone production.

## Materials and methods

### Animals

Ovaries from adult female *F. catus* (n = 104) at different stages of the natural estrous cycle during the breeding season were used. The ovaries were donated following routine spaying procedures conducted at “Centro de Salud Animal de la Municipalidad de Merlo” (Provincia de Buenos Aires, Argentina). The extracted ovaries were immediately transported to the laboratory in a refrigerated physiological solution. Those that presented stigmas, as an indication of recent ovulation, or with cysts were discarded.

### COCs isolation

Antral follicles (0.5–2 mm) were dissected from the ovary using 30-gauge needles under a stereoscopic microscope. They were punctured and their COCs were extracted in a minimum essential medium (MEM; Gibco) containing Hepes (25 mM, Gibco), L-glutamine (2 mM, Sigma-Aldrich), sodium pyruvate (1 mM, Sigma-Aldrich), penicillin/streptomycin (100 IU/mL—0.1 mg/mL, Sigma-Aldrich), and 0.3% Bovine Serum Albumin (BSA; Gibco), as previously reported (Rojo et al., 2018; Rojo et al., 2019; Jaworski et al., 2020). Only healthy COCs presenting an adequate oocyte diameter (>110 μm excluding zona pellucida) with homogeneous dark cytoplasm, complete corona radiata, and compact cumulus were collected.

### COC culture for gene expression and cumulus-oocyte expansion (C-OE) analysis

Isolated COCs (n = 237) were randomly divided and cultured in a 4-well plate in the optimal medium for C-OE analysis [MEM containing Hepes, L-glutamine, sodium pyruvate, penicillin/streptomycin, and 1% fetal bovine serum (FBS)-charcoal/stripped] previously determined empirically by our laboratory (Rojo et al., 2018). COCs were cultured in groups under different treatments containing human recombinant FSH + LH (GNTs; 10 and 5 UI/mL respectively; Merck Serono; as an inducer of C-OE and oocyte maturation), human recombinant MCP1 (10 ng/mL, Life Technologies), or in the presence of a dual EGFR inhibitor (1 μM, Lapatinib, a drug commonly used in patients; LC Labs). The concentrations used for the different treatments are based on the laboratory’s previous results, as well as the manufacturer’s datasheet and literature. COCs were cultured for 3 h for gene expression (Three separate cultures were performed, n = 78 Total COCs) or 24 h for C-OE (Five additional cultures were performed, n = 159 Total COCs) at 38°C under 5% CO_2_ in the air under different treatments as follows: a- GNT: Media + FSH + LH, b- GNT + LAPATINIB: Media + FSH + LH + EGFR Inhibitor (LAPATINIB), c- GNT + LAPATINIB + MCP1: Media + FSH + LH + LAPATINIB + MCP1, d- LAPATINIB: Media + LAPATINIB, e− LAPATINIB + MCP1: Media + LAPATINIB + MCP1, f- MCP1: Media + MCP1. The culture time for gene expression corresponds to the period in which the expression of key genes involved in C-OE (such as *HAS2*, *TNFAIP6*, *AREG*, and *PTX3*) increases against GNTs stimulation in our culture system (Rojo et al., 2018; Rojo et al., 2019; Jaworski et al., 2020). Serum was included in the media for COC due to culture serum factors being a requirement for C-OE to occur (Richards, 2005). COCs were photographed at 0 and 24 h of culture, using a digital camera attached to an Olympus microscope. At the end of the gene expression culture (3 h), COCs were stored individually at −80°C for subsequent RNA extraction and quantitative RT-PCR (qRT-PCR). On the other hand, at the end of the C-OE culture COCs were fixed in 4% paraformaldehyde for 15 min at 37°C and stored in a washing buffer (1% BSA, 0.2% powder milk, 0.2% goat serum, 0.2% donkey serum, 0.1% Triton X-100, 0.1 M glycine in 1x PBS) at 4°C for further HA IF, and medium culture was stored at −80°C for determination of steroid hormone production.

An additional set of experiments was accomplished to evaluate the effects of recombinant AREG (10 and 100 ng/mL) on the mRNA expression of *MCP1* and its receptor (*CCR2*). Healthy COCs (n = 17) isolated from antral follicles were randomly divided and cultured under different treatments using optimal conditions for gene expression as previously described (Jaworski et al., 2020) above.

### COC culture for oocyte maturation

Dissected COCs (n = 306) were randomly distributed and cultured in the optimal medium for *in vitro* maturation (IVM) used in our laboratory (MEM containing L-glutamine, sodium pyruvate, penicillin/streptomycin, and BSA), as previously reported (Jaworski et al., 2020) with slightly modifications. Oocyte maturation culture was carried out without oil since the hydrophobic characteristic of the inhibitor containing Hepes (25 mM). COCs were cultured in groups for 28 h at 38°C under 5% CO_2_ in the air under the same different treatments as follows: a- GNT, b- GNT + LAPATINIB, c- GNT + LAPATINIB + MCP1, d- LAPATINIB, e− LAPATINIB + MCP1, and f- MCP1. At the end of culture, COCs were treated briefly with hyaluronidase (1 mg/mL in MEM) to dissociate the cumulus, and naked oocytes were fixed in 4% PFA for 10 min at 37°C for indirect immunofluorescence (IF) to assess oocyte nuclear maturation, as previously described (Peluffo et al., 2010; Jaworski et al., 2020). The oocytes were then stored in washing buffer at 4°C, and medium culture was stored at −80°C for measurement of steroid hormone production.

### mRNA extraction and gene expression analysis by qRT-PCR

Total RNA was extracted from each COC (Two COCs from each group from each separate culture were extracted, total per group n = 6) using Absolutely RNA Nanoprep Kit (Agilent), as previously described (Rojo et al., 2018; Rojo et al., 2019; Jaworski et al., 2020). Briefly, DNase treatment was performed to obtain DNA-free total RNA, following the kit instructions. Afterward, 10–100 ng of RNA per sample were eluted in 12 µL of elution buffer (as measured by UV-spectrophotometer, NanoDrop 2000; Thermo Scientific). To synthesize single-stranded cDNA from total RNA, the High-Capacity cDNA Reverse Transcription Kit (Applied Biosystems) was used following the manufacturer’s instructions. The reverse transcription was performed for 2 h at 37°C using 10 μL of the RNA extracted from each COC, in a 20 μL reaction volume. The kit uses random primers as the cDNA priming method and the Multiscribe RT, a recombinant reverse transcriptase obtained from the Moloney Murine Leukemia Virus (MoMuLV). At the end of the reaction, reverse transcriptase was inactivated at 85°C for 5 min. After cDNA synthesis, qPCR for key genes within the ovulatory cascade (*AREG*, *HAS2*, *TNFAIP6*, and *PTX3*), as well as *CCR2* and *MCP1,* was conducted as previously described using the same set of primers and TaqMan probes (Rojo et al., 2018; Rojo et al., 2019; Jaworski et al., 2020). The list of primers and hydrolysis probes (TaqMan, Thermo Scientific), with the accession numbers of each target sequence, are listed in Supplementary Table S1. Relative levels of target gene expression were normalized to ribosomal RNA protein *18S* levels.

### Analyses of C-OE by HA-IF and confocal microscopy

Qualitative, as well as quantitative analyses of C-OE, focused on the change in the COC area, which is widely considered the main manifestation of this process. Because morphological assessment of expansion does not discriminate between simple loss of cell-cell contacts/death during 2-D culture and true expansion, a methodology was developed to assess a molecular indicator of C-OE: HA synthesis. A fluorescence-based technique using HA binding protein (HABP) was employed to evaluate the formation and deposition of an HA-rich ECM in expanded COCs *in vitro*, originally developed for rhesus macaque samples (Peluffo et al., 2014) with slight modifications. Briefly, an Avidin-Biotin Blocking Kit (Vector Laboratories) was used to block the endogenous signal. Subsequently, COCs were incubated with biotinylated HABP (1:200 in washing buffer; Calbiochem, Merk) for 2 h at room temperature. COCs were then washed and incubated with Cy3-labeled streptavidin (1:500 in washing buffer; Invitrogen) for 1 h at room temperature to reveal the presence of HA in the samples. After the following washing, nuclear staining was detected using Hoechst 33342 (1:1,000 in 1x PBS; Invitrogen) for 10 min at room temperature. COCs were washed, mounted on slides with glycerol/PBS solution (1:1), and coverslips affixed with clear nail polish. COCs were analyzed by confocal microscopy (Olympus FLUOVIEW FV1000 confocal laser scanning microscope) using UPLFLN 40 ×1.3 OIL CS UV objective. Full Z-stack datasets were collected for each COC, with images taken every 0.5 μm.

### Oocyte maturation and IF

To determine the nuclear maturation stage [GV-intact, germinal vesicle breakdown (GVBD), metaphase I (MI), and MII] in cat-denuded oocytes, IF was performed as previously described (Peluffo et al., 2010; Jaworski et al., 2020) due to cat oocytes having a dark appearance because of high intracellular concentration of lipids, making GV observation difficult under the optical microscope. Oocytes were briefly incubated with mouse alpha-tubulin clone DM1A (1:800 in washing buffer; Novus Biologicals) for 1 h at 37°C to detect spindle microtubules. Afterward, COCs were incubated with Alexa Fluor 488 donkey anti-mouse IgG (1:200 in washing buffer; Life Technologies) for 1 h at 37°C. DNA was labeled with Hoechst 33342 (1:1,000 in 1x PBS) for 10 min at room temperature. Oocytes were mounted on slides with glycerol/PBS solution (1:1) and coverslips affixed with clear nail polish. The maturation stage for each oocyte was analyzed and visualized using a fluorescent light microscope (Axio Imager M2, Carl Zeiss, Germany).

### Hormonal levels in culture media

Levels of estradiol (E2) and P4 in the media were measured from the different groups at 24 h (C-OE media) or 28 h (maturation media) to assess the ability to produce steroids and response under different treatment conditions. E2 and P4 levels were analyzed and expressed per well or per COC. So, the value expressed per COC corresponds to the media of that group from 6 cultures, where the raw data from each well and from each culture is divided according to the number of COC present in each well since COCs we cultured in groups. The steroids content within the culture media was analyzed by the Endocrine Laboratory at CEDIE “Hospital de Niños Ricardo Gutiérrez” using a COBAS e411 analyzer, an electrochemiluminescence-based automatic clinical platform (Roche Diagnostics GmbH) (Freire et al., 2013; Rojo et al., 2015). Intra- and inter-assay coefficients of variation of all assays were less than 4%. The minimum detectable amount of the standard for these steroids is 10 and 50 pg/mL, respectively.

### Statistical analysis

Statistical calculations were mainly performed using GraphPad Prism 5 software (GraphPad Software, Inc., San Diego, CA, United States) and SAS version 9.4 (SAS inc. NC, United States). One-Way ANOVA was used to analyze differences in gene expression, followed by Newman–Keuls for multiple comparisons with differences considered significant at *p* < 0.05. Generalized linear model, logistic regression, was used to evaluate a treatment effect on maturation to MII. The odds ratios (OR) and 95% confidence interval (CI) were calculated, and a *p*-value <0.05 was considered to be statistically significant at a significance level of 0.05.

## Results

### MCP1 was able to revert the inhibition of *AREG* mRNA by the EGFR inhibitor, but not *HAS2* or *PTX3* mRNA levels within the COC *in vitro*

**FIGURE 1:**
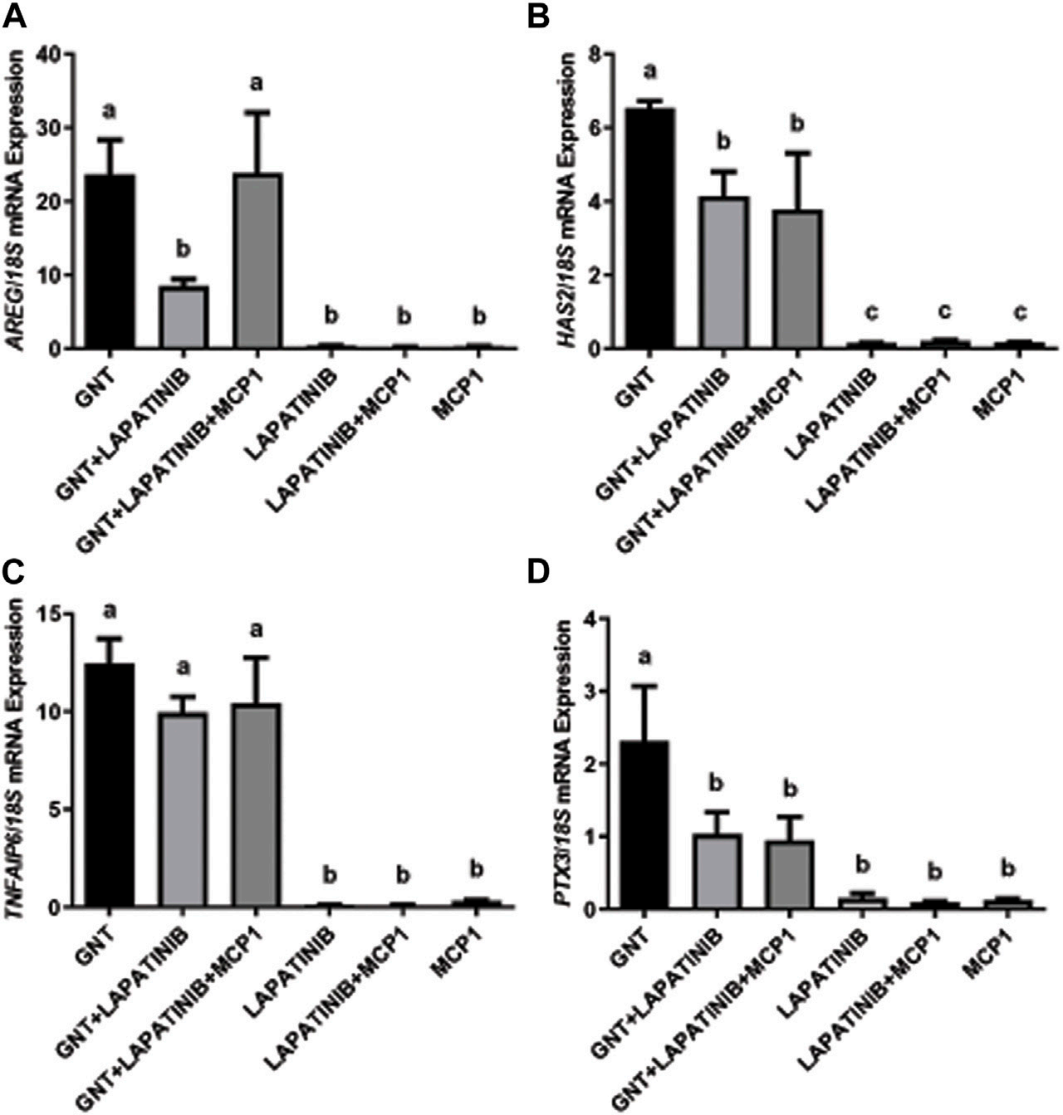
MCP1 reverted the inhibition of *AREG* mRNA by the EGFR inhibitor but not *HAS2* or *PTX3* in the feline COC *in vitro*. Normalized *AREG* **(A)**, *HAS2* **(B)**, *TNFAIP6* **(C)**, and *PTX3* **(D)** mRNA expression levels (mean ± SEM, n = 5–6 COCs per group) within the COC after 3 h of culture in the presence or absence of a positive stimulus (GNT), EGFR inhibitor (LAPATINIB), and/or the recombinant chemokine MCP1, assessed by qRT-PCR. *18S* rRNA served as the invariant control for normalization. Different letters represent significant differences between groups (ANOVA; *p* < 0.05). *AREG*: amphiregulin, *HAS2*: hyaluronan synthase 2, *TNFAIP6*: tumor necrosis factor alpha-induced protein 6, *PTX3*: pentraxin 3.

To evaluate the participation and interactions in the ovulatory cascade of the EGFR/AREG and the CCR2/MCP1 signaling pathways, we first performed COC cultures to analyze the mRNA expression of periovulatory key genes under different treatments (Figure 1). The normalized mRNA expression assessed by qRT-PCR showed that the EGFR inhibitor was able to significantly (*p* < 0.05) prevent or interfere with the GNT stimulation of *AREG* (Panel A), *HAS2* (Panel B), and *PTX3* (Panels D) mRNA expression. Interestingly, MCP1 was able to revert (*p* < 0.05) the inhibition of *AREG* mRNA expression trigged by the EGFR inhibitor (Panel A). In contrast, MCP1 could not revert (*p* > 0.05) the inhibition of *HAS2* and *PTX3* mRNA levels (Panels B and D, respectively). Regarding the mRNA expression of *TNFAIP6*, no significant differences (*p* > 0.05) were observed among the GNT, GNT + LAPATINIB, and GNT + LAPATINIB + MCP1 groups.

**FIGURE 2:**
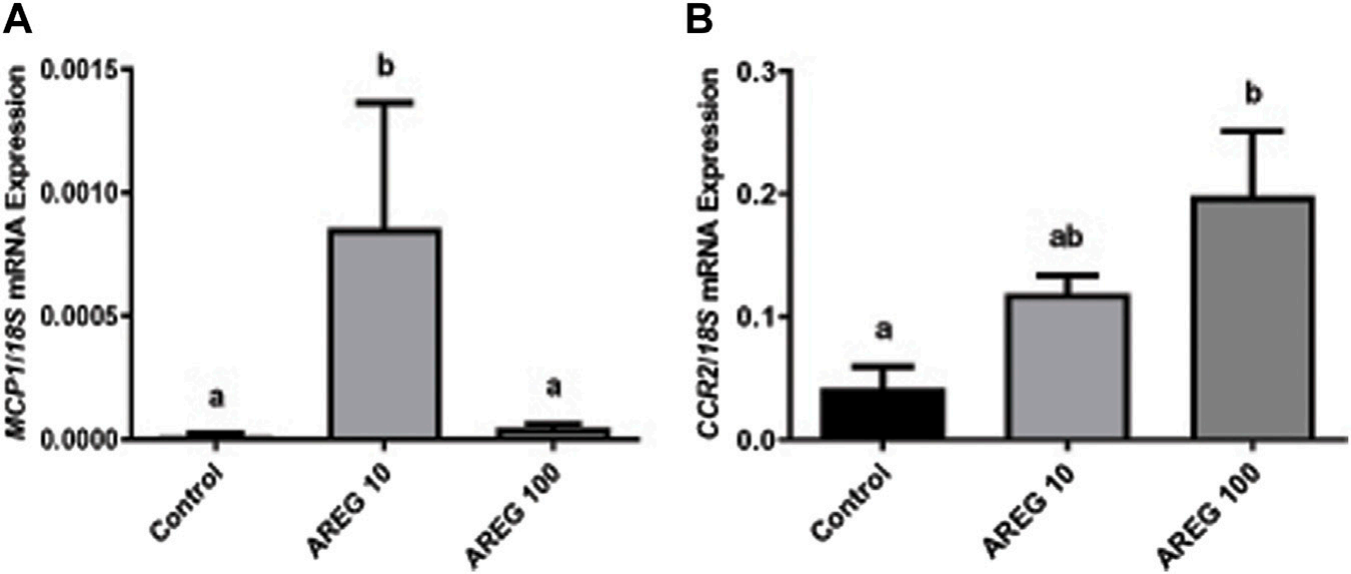
The mRNA expression of *MCP1* and *CCR2* significantly increased within the COC in the presence of recombinant AREG. Normalized *MCP1* [Panel **(A)**], and *CCR2* [Panel **(B)**], mRNA expression levels (mean ± SEM, n = 5–6 COCs per group) within the COC after 3 h of culture in the presence or absence of recombinant AREG (10 or 100 ng/mL), assessed by qRT-PCR. *18S* rRNA served as the invariant control for normalization. Different letters represent significant differences between groups (ANOVA; *p* < 0.05). AREG: amphiregulin, *MCP1*: monocyte chemoattractant protein-1, *CCR2*: chemokine CC-motif receptor type 2.

In addition, as detailed in the M&M section, a subset of the COC culture was performed to assess the mRNA expression of *MCP1* and its receptor (*CCR2*) in the presence of two different concentrations (10 and 100 ng/mL) of recombinant AREG (Figure 2), showing that AREG (10 ng/mL) significantly stimulated the mRNA levels of the feline chemokine *MCP1* in comparison to the control (Panel A). Whereas AREG significantly increased the *CCR2* mRNA levels at the higher concentration (100 ng/mL, Panel B).

### MCP1 reverted the inhibition of GNT-stimulated HA synthesis triggered by the EGFR inhibitor within the expanded matrix in the COC *in vitro*

**FIGURE 3:**
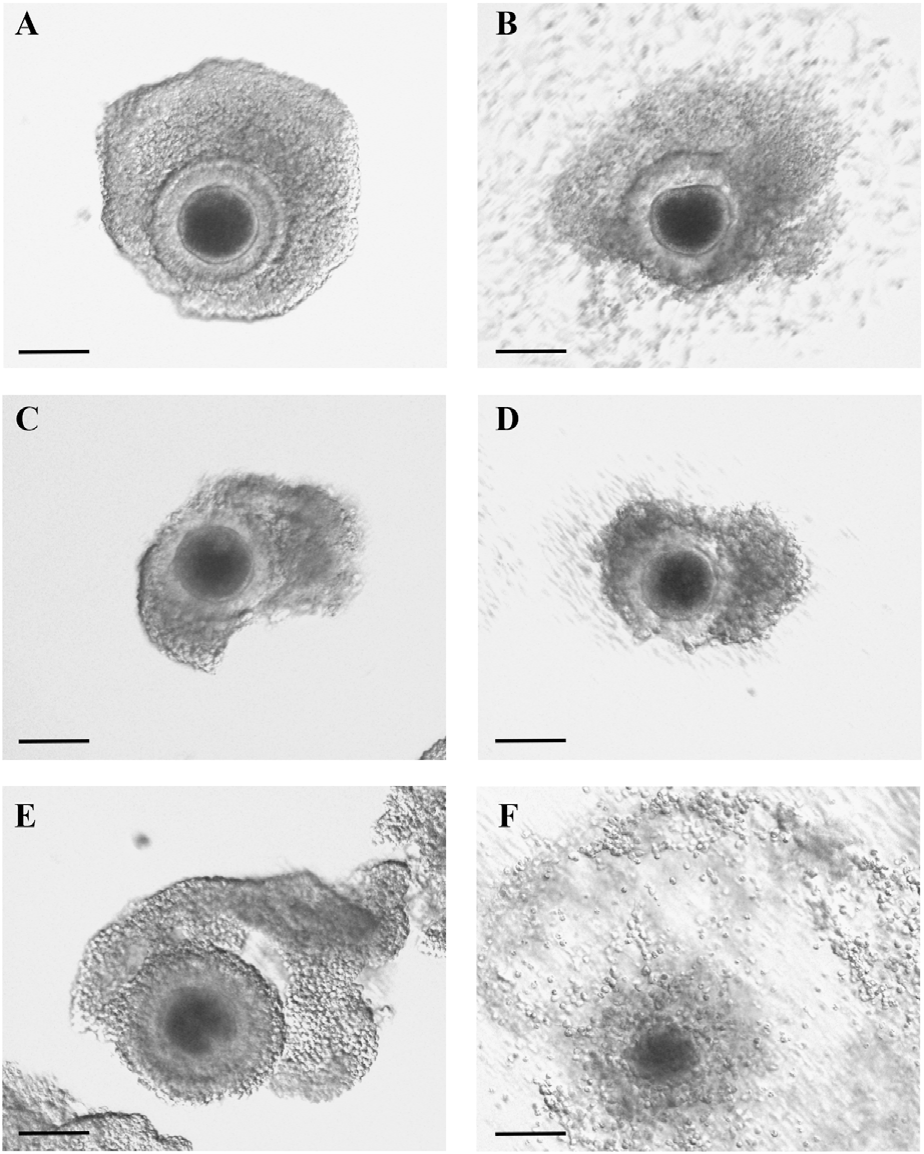
MCP1 restored C-OE inhibited by the EGFR inhibitor. Representative images of feline COCs from C-OE culture were taken prior to **(A,C,E)** and at the end of culture 24 h; **(B,D,F)**. COCs were incubated (24 h) with GNT **(A–B)**, GNT + LAPATINIB **(C–D)**, GNT + LAPATINIB + MCP1 **(E–F)**. Scale bars 100 μm.

**FIGURE 4:**
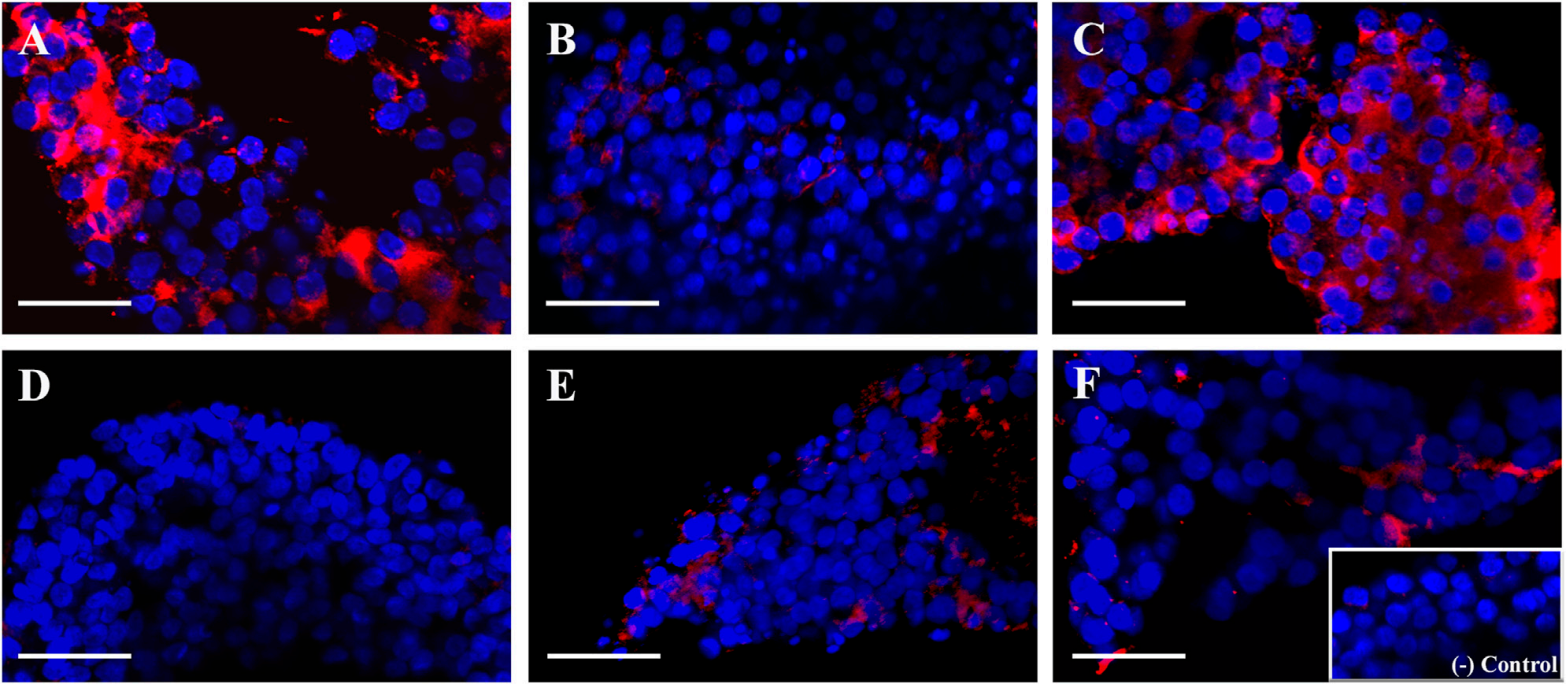
MCP1 restored the hyaluronic acid (HA) synthesis inhibited by the EGFR inhibitor. Representative confocal microscopy images (x 40) of feline COCs from C-OE culture under different treatments after HA IF staining. COCs were incubated (24 h) with GNT **(A)**, GNT + LAPATINIB **(B)**, GNT + LAPATINIB + MCP1 **(C)**, LAPATINIB **(D)**, LAPATINIB + MCP1 **(E)**, MCP1 **(F)**. **(A–F)** Nuclear staining is displayed in blue (Hoechst 33342), and HA in red (Cy3). No staining was observed in the negative control (in the absence of the HA binding protein, Insert). Scale bars 50 μm.

After evaluating the mRNA expression of periovulatory key genes, studies were conducted to evaluate the effect of the EGFR inhibitor on feline C-OE triggered by GNTs (as an inducer of C-OE), in the presence or absence of recombinant MCP1 *in vitro*. As a first approach, morphological features of C-OE were analyzed under the inverted microscope 24 h after culture (Figure 3) showing that the EGFR inhibitor blocked the C-OE in comparison to the GNT group. Interestingly, the addition of MCP1 seemed to restore the C-OE. To confirm these, we assessed the main molecular indicator of C-OE, the HA synthesis, by IF. Representative confocal images of feline COCs showing the formation and deposition of an HA-rich ECM in expanded COCs 24 h after culture under different conditions are depicted in Figure 4. IF performed in fixed COCs using a HA binding protein confirmed that GNT-stimulated HA synthesis within the expanded matrix in the COC (Panel A) was inhibited in the presence of the EGFR inhibitor (Panel B). In contrast, MCP1 could revert this effect, and HA was observed throughout the whole COC (Panel C). The LAPATINIB control group showed minimal HA-positive staining (Panel D). In contrast, all the groups containing the recombinant MCP1 consistently showed synthesis, and secretion of HA throughout and among all COCs analyzed (Panels C, E, F), being the group GNT + LAPATINIB + MCP1 the one with the strongest HA staining observed (Panel C). Supplementary Figure S1 depicts representative confocal microscopy images from three separate cultures of feline COC from C-OE culture under different treatments after HAIF staining.

### High percentage of feline MII-stage oocytes observed in the presence of GNT, MCP1, and the EGFR inhibitor

**FIGURE 5:**
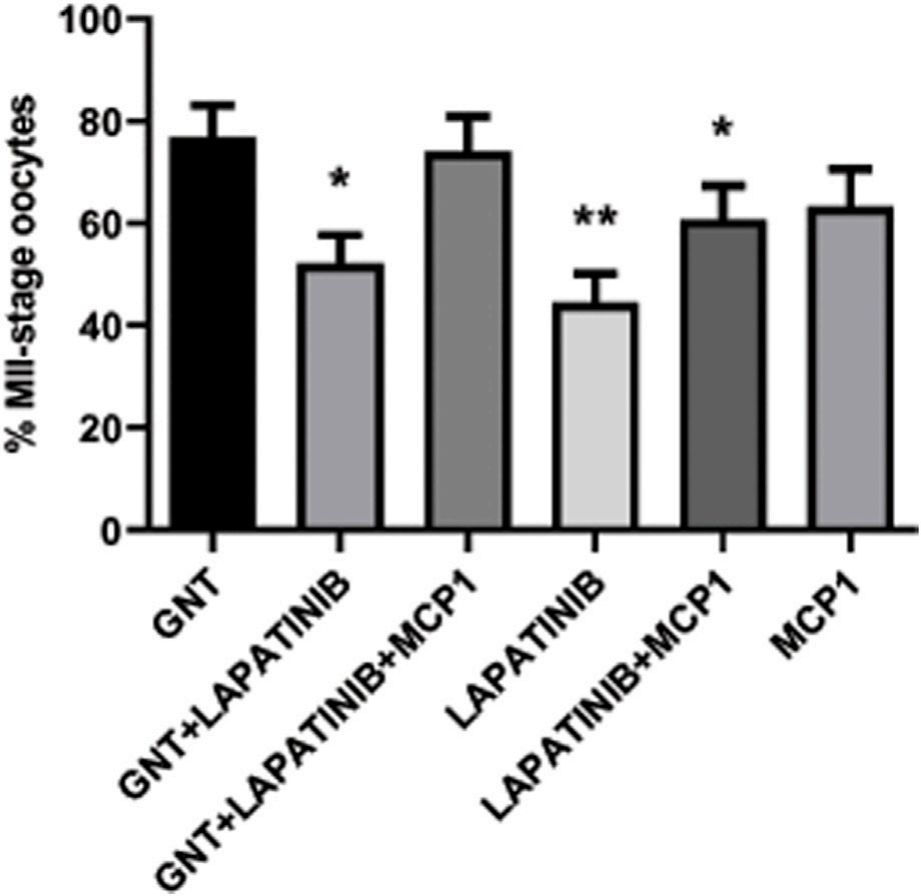
*In vitro* feline oocyte maturation under different treatment conditions. Proportion of metaphase II (MII) stage oocytes after 28 h in culture, in the presence or absence of a positive stimulus (GNT), a dual EGFR inhibitor (LAPATINIB), and/or recombinant chemokine MCP1, assessed by IF (expressed as mean percentage ±SEM, n = 306 total COCs). Generalized linear model, logistic regression, was used to evaluate a treatment effect on maturation to MII. * *p*-value <0.05 compared to GNT; ** *p*-value <0.001 compared to GNT.

Oocyte maturation was measured as the percentage of oocytes that reached the MII stage after 28 h in culture under different treatment conditions. As expected, a high percentage of MII-stage oocytes was observed in the presence of GNT. Interestingly, the addition of the EGFR inhibitor to the media indeed significantly decreased (*p* = 0.0135) this stimulation (Figure 5), as expected based on other species. In contrast, the addition of MCP1 could revert this inhibition, leading to a recovery with an increased percentage of MII-stage oocytes and returning the percentage of MII-stage oocytes to a similar percentage obtained in the presence of GNT (GNT vs. GNT + LAPATINIB + MCP1, *p* = 0.7463).

The EGFR inhibitor alone did not affect oocyte maturation and the percentage of MII oocytes is significantly lower in comparison to the GNT (*p* = 0.0009). The percentage of MII oocytes within this group is like unstimulated COCs (spontaneous maturation) that usually represent around 30%–50%.

### MCP1 restored *in vitro* P4 production inhibited by the EGFR inhibitor in C-OE cultures

To further study the possible effect and interactions of the EGFR/AREG and the CCR2/MCP1 signaling pathways, we assessed the E2 and P4 production by the COCs under different treatments. Levels of E2 and P4 measured in the culture media from the different groups are detailed in Table 1 (Panel A: C-OE media; Panel B: IVM media).

**TABLE 1:** Steroid levels present in the media of feline COC after 24 (Panel A: C-OE) or 28 (Panel B: IVM) hours in culture under the different treatments. E2 and P4 levels were analyzed and expressed per well or per COC (n = 6 experiments).

(A)						
Treatment group	E2				P4	
	(per well)		(per COC)		(per well)	(per COC)
GNT	13.12 ± 0.86^a^		2.55 ± 0.33^a^		115.75 ± 52.46^a^	22.95 ± 10.62^a^
GNT + LAPATINIB	14.22 ± 1.00^a^		2.75 ± 0.35^a^		<50	NA
GNT + LAPATINIB + MCP1	14.18 ± 0.85^a^		2.61 ± 0.32^a^		133.50 ± 42.50^a^	29.20 ± 8.62^a^
LAPATINIB	<10		NA		<50	NA
LAPATINIB + MCP1	<10		NA		<50	NA
MCP1	<10		NA		<50	NA

(B)						
Treatment group	E2				P4	
	(per well)		(per COC)		(per well)	(per COC)
GNT	20.38 ± 2.21^a^		2.51 ± 0.65^a^		112.80 ± 17.65^a^	12.68 ± 1.65^a^
GNT + LAPATINIB	17.36 ± 2.03^abc^		2.36 ± 0.45^a^		93.00 ± 11.23^a^	14.05 ± 2.13^a^
GNT + LAPATINIB + MCP1	18.38 ± 2.40^ab^		2.02 ± 0.26^a^		93.60 ± 11.72^a^	14.87 ± 1.93^a^
3;LAPATINIB	10.90 ± 0.78^c^		1.53 ± 0.42^a^		100.80 ± 14.06^a^	8.10 ± 0.98^a^
LAPATINIB + MCP1	13.00 ± 0.96^bc^		1.83 ± 0.32^a^		100.70 ± 8.58^a^	9.56 ± 2.09^a^
MCP1	12.51 ± 0.94^bc^		1.42 ± 0.27^a^		109.80 ± 16.40^a^	7.87 ± 1.76^a^

Values are the mean ± SEM (pg/mL) of E2 and P4. Different letters represent significant differences among groups within the same column (ANOVA; *p* < 0.05). Limit of detection: E2 (<10 pg/mL) and P4 (<50 pg/mL). GNT: gonadotropins; LAPATINIB: EGFR, inhibitor; NA: not applicable.

Steroids production at 24 h culturing the COCs within the C-OE media was detected only in groups containing GNTs (Panel A). EGFR inhibitor did not have a significant effect on E2 production. However, the presence of this inhibitor in the media blocked the P4 production stimulated by GNT, with no detectable levels observed within this group. But interestingly, MCP1 was able to restore the P4 production. Steroid data was analyzed (and expressed) by total levels detected per well but also, per COC considering that COCs were cultured in groups. Similar results were observed among groups when data were analyzed either per well or per COC.

Panel B shows the results from the IVM media. All samples from IVM media showed E2 and P4 levels with the highest concentrations observed within the GNT groups when analyzing per COC. In the IVM media, the EGFR inhibitor did not significantly affect the production of these steroids triggered by GNT (*p* > 0.05).

## Discussion

The present study reveals critical roles and interactions of the EGFR/AREG and the CCR2/MCP1 signaling pathways in the ovulatory cascade. We observed that MCP1 was able to revert the inhibition of *AREG* mRNA expression by an EGFR inhibitor within the feline COC. In accordance, we also observed that the GNT-stimulated HA synthesis and P4 production, blocked by the EGFR inhibitor, was recovered by the addition of recombinant MCP1 in the C-OE culture media. Regarding oocyte nuclear maturation, recombinant MCP1 could also revert the inhibition triggered by the EGFR inhibitor, leading to a recovery in the percentage of MII-stage oocytes.

As a first approach, we studied the interactions between the EGFR/AREG and the CCR2/MCP1 signaling pathways within the mRNA expression of periovulatory key genes (*AREG*, *HAS2*, *TNFAIP6*, and *PTX3*). As expected, the EGFR inhibitor was able to prevent or interfere with the GNT stimulation of *AREG*, *HAS2*, and *PTX3* mRNA expression, but not with *TNFAIP6*. Park et al. (2004) demonstrated that LH stimulation induced the transient and sequential expression of the EGF family members such as AREG, and these EGF-related growth factors act as paracrine mediators that propagate the LH signal throughout the follicle to promote periovulatory events in both oocytes and cumulus cells. Also, it was demonstrated by others that EGFR activity was essential for periovulatory events (such as oocyte maturation and cumulus expansion) using pharmacological inhibitors and genetic disruption to the EGF network (Ashkenazi et al., 2005; Hsieh et al., 2007). Thus, we expected that the inhibition of the EGFR within the feline COC would have a significant effect on the periovulatory genes, as observed. Surprisingly, MCP1 was only able to revert the inhibition of *AREG* mRNA triggered by the EGFR inhibitor. We previously demonstrated that a highly selective antagonist of CCR2 (the main receptor of MCP1) significantly repressed the stimulation of *AREG*, *HAS2*, and *TNFAIP6* mRNA levels triggered by AREG within the feline COC (Jaworski et al., 2020), suggesting that CCR2/MCP1 system was downstream the EGF-related factors in the ovulatory cascade. On the other hand, we observed in the current study that AREG significantly stimulated the mRNA levels of the chemokine *MCP1* and its receptor *CCR2* within the feline COC. Considering that we observed that AREG stimulated directly *CCR2* and *MCP1* mRNA, and MCP1 stimulated *AREG* mRNA (Rojo et al., 2019), we could think about the existence of a positive loop within EGFR/AREG and CCR2/MCP1 pathways, that maybe guarantees and propagate the periovulatory process triggered by LH. With regard to the mechanism involved in regulating MCP1 expression by the EGFR pathway, it was reported that the EGFR signaling pathway can activate the PKC and MAPKs (Rabinovitz et al., 2004; Frijns et al., 2010), which will then induce the activation and translocation of nuclear factor (NF) κb to the nucleus. And it has been shown that induced expression of MCP1 is strongly dependent on the activation of the transcription factor NFκb (Ueda et al., 1994; Ueda et al., 1997).

Previously, our laboratory has demonstrated that a highly selective CCR2 antagonist significantly decreased the GNT stimulation of *PTX3* mRNA expression within the feline COC; however, it could not inhibit the stimulation of *PTX3* mRNA levels induced by AREG (Jaworski et al., 2020), indicating that PTX3 stimulation by AREG is independent of the CCR2/MCP1 pathway. In that manuscript, we proposed that CCR2/MCP1 could act by modulating PTX3 indirectly through the induction/stimulation of AREG. Mainly because PTX3 plays a role in cumulus matrix stability (Salustri et al., 2004), which may be a later event in the matrix formation, while HAS2 and TNFAIP6 play an earlier role. Thus, the current results, showing that MCP1 could not revert the inhibition, support this idea. On the other hand, *HAS2* mRNA expression surprisingly was not recovered by the presence of the recombinant MCP1. Based on our previous work that showed that the CCR2 antagonist significantly decreased the stimulation of *HAS2* mRNA levels triggered by AREG within the feline COC (Jaworski et al., 2020), we had expected that MCP1 was going to revert the significant decrease triggered by the EGFR inhibitor. Furthermore, in the present study, the addition of recombinant MCP1 reversed the reduction of the GNT-stimulated HA synthesis within the COC triggered by the EGFR inhibitor. One possible explanation for this discrepancy could be that in the groups containing GNT + LAPATINIB, and GNT + LAPATINIB + MCP1 there was high variability within the *HAS2* mRNA expression. High variability in the HA-IF was also observed in the GNT + LAPATINIB group, where we noted samples with a total blockage of HA synthesis while others with some HA-positive staining. So, this variability in the response to the inhibitor within the different COCs could eventually explain this discrepancy. Interestingly, in all the groups with MCP1, the presence of HA was always detected among the cumulus cells. In agreement with these observations, MCP1 was shown to directly stimulate *HAS2* mRNA within the feline COC (Rojo et al., 2019). Regarding *TNFAIP6*, as the EGFR inhibitor did not have a significant effect on its mRNA levels, no further analysis comparing the presence of MCP1 is worth doing. Why the EGFR inhibitor did not have a significant effect on *TNFAIP6* mRNA could be explained by the fact that GNT stimulation of TNFAIP6 depends mainly on the PGE2 pathway (Ochsner et al., 2003).

EGFR is a member of the ErbB family of RTKs, which is composed of 4 receptors (named ErbB1-4) (Herbst, 2004), and all of them have been detected in the ovary (Richani and Gilchrist, 2018). For example, these 4 transcripts have been observed in human preovulatory follicle granulosa and cumulus cells following hCG stimulation (Zamah et al., 2010). Whereas three of them (Erb1-3) have been shown to be constitutively expressed in mouse granulosa and cumulus cells (Noma et al., 2011; Richani and Gilchrist, 2018). Since we used a dual EGFR inhibitor (Lapatinib), that also inhibits ErbB2, we cannot discard that the effect observed was exclusively from the EGFR (also known as ErbB1) inhibition. ErbB2, has no known ligand, but it can dimerize with other ErbB receptors (Citri and Yarden, 2006). For example, ErbB3 requires heterodimerization with ErbB2 to become active after its ligands (neuregulins, NGR, 1 and 2) bind since it lacks tyrosine kinase activity (Citri and Yarden, 2006). But even though the EGF family comprises 11 proteins with highly similar functional and structural properties (Schneider and Wolf, 2009), in the preovulatory follicle only some of these members seem to be relevant, mainly AREG. And since AREG signals by binding EGFR only, it is very likely that the repression in the periovulatory genes observed in the presence of the EGFR inhibitor is due to the blockage of EGFR activation. For example, in rhesus macaque, the mRNA expression of *AREG* and *EREG* significantly increased in the preovulatory follicle after an ovulatory stimulus, whereas the *BTC* mRNA was minimal and did not vary throughout the periovulatory interval (Xu et al., 2011). Moreover, the mRNA expression of other EGF family members was minimal or undetectable in the primate preovulatory follicle (Xu et al., 2011). Furthermore, one of the authors demonstrated a rapid and significant increase in the AREG protein content in the primate follicular fluid in agreement with the mRNA data (Peluffo et al., 2012). In humans, higher levels of the AREG protein content than EGF were observed in the follicular fluid of women undergoing IVF protocols, having a concentration of around 100 ng/mL in comparison with 5 pg/mL, respectively (Inoue et al., 2009). Moreover, EREG and BTC peptides have not been distinguished in human follicular fluid or conditioned media from mouse COC (Zamah et al., 2010; Richani et al., 2013). In the domestic cat, NRG2 and ErbB2 transcript expression were the transcripts observed from the EGF family in the preantral follicles, with a sequential decrease of NRG2 expression from primordial to secondary follicles (Kehoe et al., 2021). Thus, even though we cannot completely discard the involvement of ErbB2, the evidence supports that the effects observed by the EGFR inhibitor are likely due to the blockage of EGFR activation.

Regarding oocyte maturation, the EGFR inhibitor significantly decreased the percentage of MII-stage oocytes in comparison to the GNT group, as expected. Based on the literature, where several papers have reported positive effects of EGF on oocyte maturation in different species including; women, cats, dogs, cows, pigs, sheep, horses, and mice (Rieger et al., 1998; De La Fuente et al., 1999; Guler et al., 2000; Procházka et al., 2000; Lorenzo et al., 2001; Gomez et al., 2003; Merlo et al., 2005; Cui et al., 2006). Interestingly, MCP1 was able to restore it. This result is in accordance with our previous data that showed a stimulatory effect of MCP1 on meiosis (Jaworski et al., 2020), but it also confirmed that CCR2/MCP1 signaling pathway is downstream EGFR/AREG pathway within the ovulatory cascade. Activation of the CCR2 signaling includes Ca^2+^, PKC, MAPK, and Rho GTPase (Sozzani et al., 1991; Yen et al., 1997; Ashida et al., 2001; Jones et al., 2003). Interestingly, the chemokine MCP1 has been shown to also reduce the accumulation of cAMP following stimulation of adenylate cyclase with forskolin (O’Boyle et al., 2007). Thus, the binding of MCP1 to its receptor triggers two main events that play a critical role in the re-initiation of meiosis, such as inhibition of the adenylate cyclase and stimulation of calcium influx. On the other hand, it is important to emphasize that EGFR inhibitor did not provoke a complete blockage on the oocyte maturation stimulated by GNT, as it did with the synthesis of HA and the production of P4, supporting the idea that GNT stimulation triggered different pathways together with the EGFR. It is important to appreciate that during the ovulatory cascade, multiple signaling pathways are activated and required to trigger all the necessary events that *in vitro* are hard to mimic. Since the GNT + LAPATINIB + MCP1 group was one of the groups with the highest percentage of MII-stage oocytes, this treatment could provide an additional good option for IVM in fertility clinics. However, further studies are needed to assess the oocyte developmental outcome of this treatment *in vitro*. Especially, as Richani and Gilchrist highlighted, EGF-like peptide signaling in granulosa and cumulus cells was critical not only for oocyte meiotic maturation but also for this signaling network coordinated oocyte cytoplasmic maturation, influencing the developmental capacity of the oocyte (Richani and Gilchrist, 2018).

E2 and P4 levels were assessed and detected in the media from COCs cultured under different treatments in both culture systems (C-OE and IVM). Steroid production at 24 h from the C-OE media was detected only in groups containing GNTs. Interestingly, the presence of the EGFR inhibitor blocked the P4 production stimulated by GNT, with MCP1 being able to restore its production by the COCs. In contrast, EGFR inhibitor did not have a significant effect on E2 levels. Intriguingly, it was reported that EGF-related factors mediate the GNT action through the induction of steroid and prostaglandin production (Jamnongjit et al., 2005; Shimada et al., 2006). It is known that P4 is essential for the normal development of periovulatory events, especially for follicular rupture (Gaytan et al., 2004). Moreover, P4 produced by the COC seems to play a role in oocyte nuclear maturation (Shimada and Terada, 2002). In rhesus macaques, for example, a P4 agonist treatment can promote oocyte maturation to the MII-stage, even in the absence of the LH surge (Borman et al., 2004). And more importantly, Tian et al. (2017) demonstrated that P4 from cumulus cells promotes oocyte developmental potential in mice. So, it is not surprising that the EGFR inhibitor completely blocked its production by GNT as it is known to be a paracrine mediator of the LH surge. But it is interesting and novel that the chemokine MCP1 did revert that. P4 synthesis and release from the cumulus cells during the ovulatory cascade are believed to have another important action. Based on the sperm chemotaxis model, the spermatozoa can sense a gradient of P4 derived from the cumulus cells surrounding the oocyte (after ovulation) and swim toward the source of P4 (Villanueva-Diaz et al., 1995). Thus, our current results demonstrate the interaction between EGFR and CCR2 signaling pathways in the periovulatory process (including P4 production) and they support a direct role for chemokines like MCP1 in regulating events necessary to ovulate a fertilizable oocyte. In agreement with these observations, rodent studies suggest that chemokine signaling regulates the assembly of the cumulus extracellular matrix and thus fertilization (Tamba et al., 2008). Also, it was reported that when PGE2 binds to its receptor subtype 2 (PTGER2) its actions were mediated by some chemokines in mice (Yodoi et al., 2009).

Regarding the steroid production of COC during IVM, the inhibitor did not significantly affect the production of E2 or P4 by the COCs. Although the highest concentrations were observed within the GNT and GNT + LAPATINIB + MCP1 groups. In this work, we demonstrated that feline cumulus cells can produce E2 and P4 in response to a stimulus using either of our culture systems (C-OE and IVM), with detectable levels in the culture media. In the eighties, McNatty et al. (1980) has shown that human COCs from antral follicles were steroidogenically competent units (capable of synthesizing progesterone, androgens, and estrogens) with the capacity to modify the endocrine microenvironment of the follicle. P4 produced by the COC was reported to participate in the optimal milieu within the follicle (Shimada and Terada, 2002). Based on our results, we believe that cumulus cells previously incorporated the precursors for the synthesis of these steroid hormones; although dedifferentiation of cumulus cells to granulosa cells cannot be discarded. Discrepancies in E2 and P4 values between the C-OE and IVM culture systems may be due to the differences in time (28 h vs. 24 h), the presence of FBS-charcoal vs. BSA, or differences in concentrations of GNT that were optimal for each system.

**FIGURE 6:**
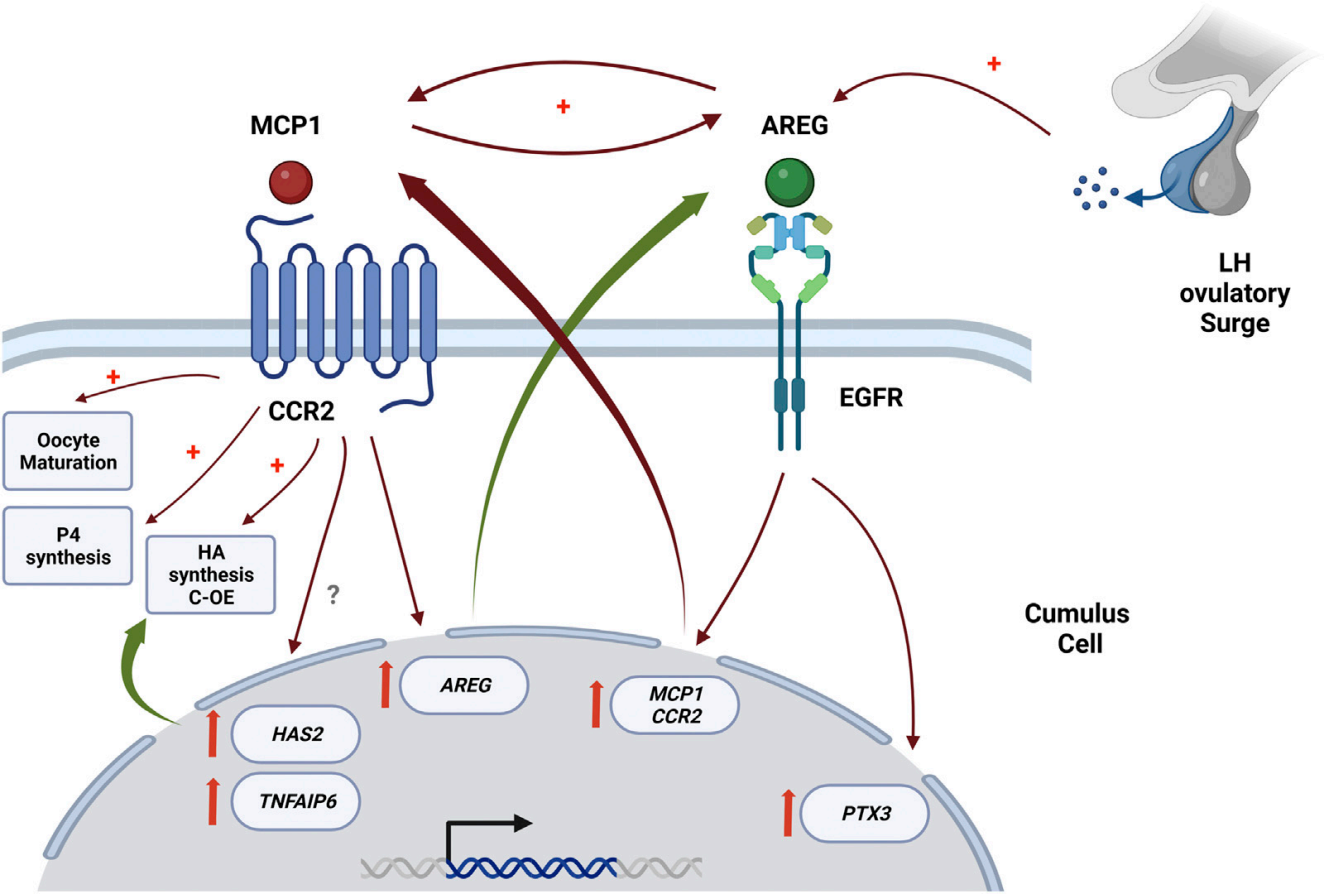
Proposed interaction between CCR2 and EGFR pathways in the C-OE process and oocyte maturation, with CCR2/MCP1 signaling pathway being downstream of the EGFR in the ovulatory cascade. This scheme is proposed based on mainly our current results in conjunction with some of our previously published results (Rojo et al., 2019; Jaworski et al., 2020). This figure was creating in Biorender.com.

In summary, our results confirm the chemokine receptor CCR2 as a novel intermediate in the ovulatory cascade and demonstrate that the EGFR/AREG and the CCR2/MCP1 signaling pathways play critical roles in regulating feline C-OE and oocyte nuclear maturation, with CCR2/MCP1 signaling pathway being downstream EGFR/AREG pathway within the ovulatory cascade. Considering that AREG stimulates directly *CCR2* and *MCP1* mRNA, and MCP1 stimulates *AREG* mRNA, it is likely the existence of a positive loop within EGFR/AREG and CCR2/MCP1 pathways, that may guarantee and propagate the periovulatory process triggered by LH. Figure 6 shows a scheme of the proposed interaction between CCR2 and EGFR pathways in the C-OE process and oocyte maturation. Therefore, a better understanding of these novel interactions within the COC during the ovulatory cascade could aid in increasing our understanding of events required for fertilization, thereby leading to the identification of possible causes of infertility and/or novel markers for oocyte quality that would improve IVM culture conditions and help in the identification of novel non-hormonal targets for contraception, as well as.

## Data Availability

The original contributions presented in the study are included in the article/Supplementary Material, further inquiries can be directed to the corresponding author.
