# Supplementary material for: Interaction between epidermal growth factor receptor and C-C motif chemokine receptor 2 in the ovulatory cascade
Source: Front Cell Dev Biol. 2023 Apr 4;11:1161813. doi: 10.3389/fcell.2023.1161813 (PMC10110862; doi:10.3389/fcell.2023.1161813)
Supplement: Supplementary file 1 [file Table1.DOCX]

Supplementary Material

# Supplementary Table 1| Minor groove binder probe sequences and forward and reverse primers.

| **Gen Symbol** | **Probe (5´-3´) 6FAM-Sequence-MGBNFQ (1)** | **Forward (5´-3´)** | **Reverse (5´-3´)** | **Target sequence accession number** |
| --- | --- | --- | --- | --- |
| ***AREG*** | TCCATGAAGACTCACAGCATGGTTGA | TACTTTGGTGAACGGTGTGG | GCAGACACAAAGGCAGCTAT | XM_006931061.4 |
| ***HAS2*** | CACGGCTCGATCCAAGTGCC | GAGTCTGGGCTATGCAACAA | TGTACAGCCACTCTCGGAAG | XM_004000089.4 |
| ***TNFAIP6*** | CCCGCTGTCACTGAAGCGTCA | ACGGCTTTGTGGGAAGATAC | GCATCCACAGCAGCATACTT | XM_003990758.4 |
| ***PTX3*** | TGCAGGATCCCTCCCTCAGGA | CATGTCCTTGTGGGTAAACG | TTCATCAAAGCCACCACCTA | XM_023260440.1 |
| ***MCP1*** | TCACCAGCAGCAAGTGTCCCAA | AAGATCTCGATGCAGAGGCT | GGGTCTTCTTGTCCAGGTGT | XM_003996556.4 |
| ***CCR2*** | CTGTTGCATCAACCC | GACGGAGACCCTGGGAATG | AACACGGAGAGATGCCTTCTG | XM_006928639.2 |
| ***18S*** | CAGCAGGCGCGCAAATTACCCA | CGGCTACCACATCCAAGGAA | GGGCCTCGAAAGAGTCCTGT | XR_002740181.1 |

(1) For *18S*: Probe (5’-3’) VIC-Sequence-MGBNFQ [MGB (minor groove binder) probes incorporate a 5' reporter and a 3' nonfluorescent quencher (NFQ)]

*AREG*: amphiregulin, *HAS2*: hyaluronan synthase 2, *TNFAIP6*: tumor necrosis factor alpha-induced protein 6, *PTX3*: pentraxin 3, *MCP1*: monocyte chemoattractant protein-1, *CCR2*: C-C motif chemokine receptor 2.
