# Supplementary figures and images for: Interaction between epidermal growth factor receptor and C-C motif chemokine receptor 2 in the ovulatory cascade
Source: Front Cell Dev Biol. 2023 Apr 4;11:1161813. doi: 10.3389/fcell.2023.1161813 (PMC10110862; doi:10.3389/fcell.2023.1161813)

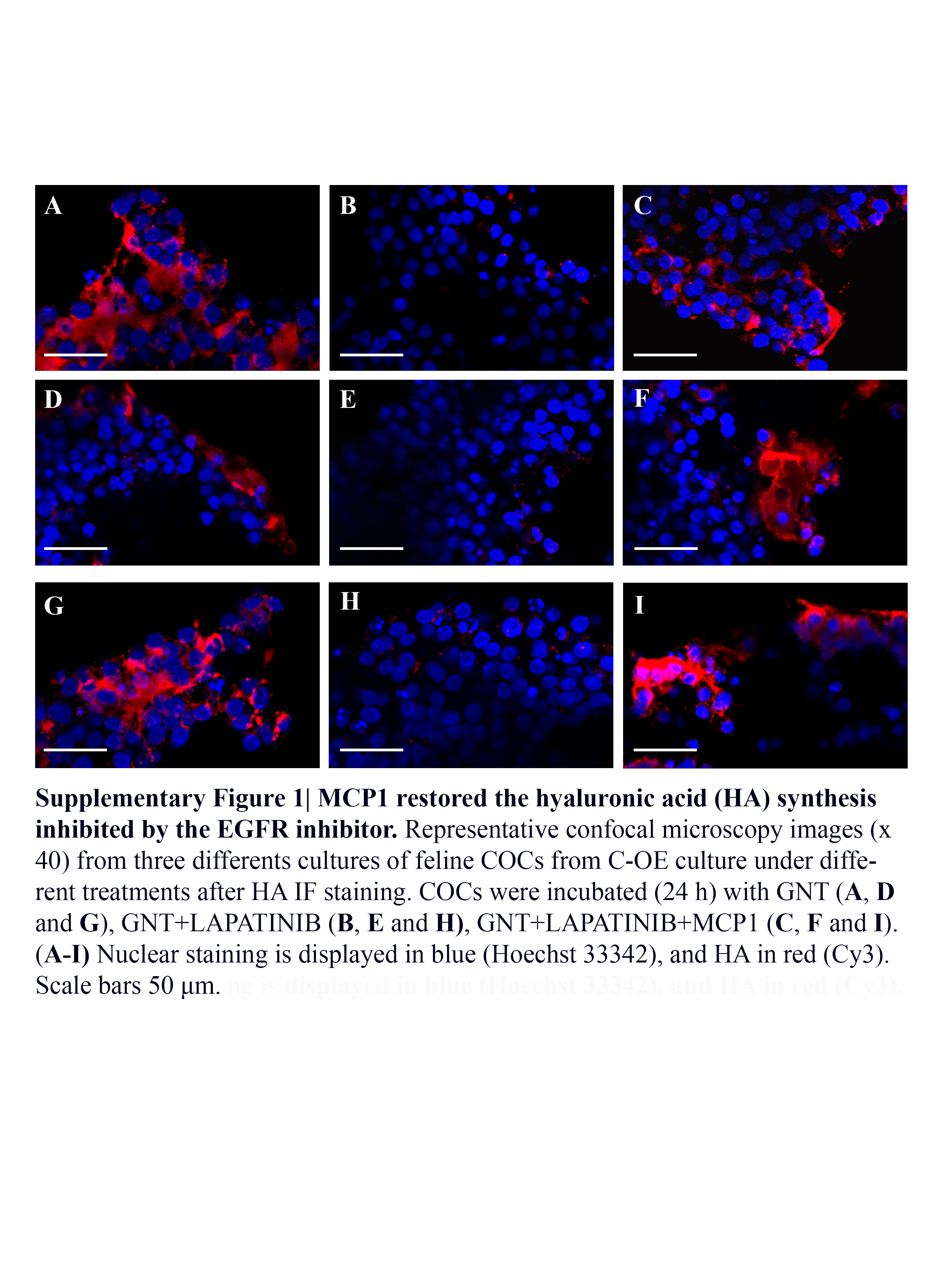

Supplement: Supplementary file 2 [file Image1.TIF]
